# Sulfated Chitosan Induces Membrane Disruption, Aggregation, and Antibiofilm Activity in *Piscirickettsia salmonis*: A Biomimetic Strategy as an Antimicrobial Alternative in Aquaculture

**DOI:** 10.3390/antibiotics15050435

**Published:** 2026-04-27

**Authors:** Darwuin Arrieta-Mendoza, Alejandro A. Hidalgo, Andrónico Neira-Carrillo, Sergio A. Bucarey

**Affiliations:** 1 Centro Biotecnológico Veterinario, Biovetec, Departamento de Ciencias Biológicas Animales, Facultad de Ciencias Veterinarias y Pecuarias, Universidad de Chile, Santa Rosa 11735, La Pintana, Santiago 8820000, Chile; 2 Programa de Doctorado en Ciencias Silvoagropecuarias y Veterinarias, Facultad de Ciencias Agronómicas, Campus Sur, Universidad de Chile, Santa Rosa 11315, La Pintana, Santiago 8820808, Chile; 3 Catedra de Farmacología y Toxicología, Departamento de Ciencias Biomédicas, Facultad de Ciencias Veterinarias, Universidad Central de Venezuela, Avenida Universidad, vía El Limón, Campus Maracay, Maracay 2105, Venezuela; 4 Escuela de Química y Farmacia, Facultad de Medicina, Universidad Andres Bello, Sazié 2320, Santiago 8360000, Chile; 5 Laboratorio Polyforms, Departamento de Ciencias Biológicas Animales, Facultad de Ciencias Veterinarias y Pecuarias, Universidad de Chile, Santa Rosa 11735, La Pintana, Santiago 8820000, Chile

**Keywords:** sulfated chitosan, *Piscirickettsia salmonis*, antibiofilm activity, antimicrobial alternative, heparan sulfate mimetic, bacterial membrane disruption, bacterial aggregates

## Abstract

**Background:** Sulfated chitosan (ChS) is a chemically modified polysaccharide derived from chitin that mimics heparan sulfate (HS) structures and has emerged as a promising antimicrobial biomaterial. *Piscirickettsia salmonis*, the etiological agent of Salmonid Rickettsial Septicemia (SRS), represents the main driver of antibiotic use in Chilean aquaculture. **Objective:** In this study, the in vitro antibacterial activity of ChS against *P. salmonis* was evaluated. **Methods:** Elemental characterization by SEM-EDS and FTIR analysis confirmed successful sulfation of the polymer, with a degree of sulfation ranging from 0.92 to 0.95. Additionally, X-ray diffraction (XRD) analysis revealed a reduction in polymer crystallinity, indicating a transition toward a more amorphous structure associated with increased molecular flexibility and functional group accessibility. **Results:** Antibacterial assays revealed a minimum inhibitory concentration (MIC) of 1500 µg/mL and a minimum bactericidal concentration (MBC ≥ 1500 µg/mL). LIVE/DEAD™ fluorescence imaging showed the formation of bacterial aggregates with increasing size, frequency, and red fluorescence compared to controls over the exposure to ChS, indicating progressive membrane damage. This was supported by a reduction (*p* < 0.05) in the Green/Red fluorescence ratio of 37–46% between 5 h and 96 h of exposure, corresponding to alteration of the cell membrane. Scanning electron microscopy revealed pronounced morphological alterations by ChS, including surface disruption and loss of cellular integrity. This was more severe compared to commercial chitosan (ChC). Also, ChS reduced (*p* < 0.05) biofilm formation (>50% at day 6 and 34.8% at day 8). **Conclusions:** These results demonstrated that ChS disrupts the cell membrane and reduces biofilm formation in *P. salmonis*, thereby affecting viability. This is the first report of the antibacterial effect of ChS, an HS analogue, against *P. salmonis*.

## 1. Introduction

Chile is the world’s second-largest producer and exporter of salmon, second only to Norway [1]. This places Chile in a significant position within the global salmon industry. *Piscirickettsia salmonis* is the causative agent of salmonid rickettsial syndrome (SRS), the bacterial disease with the highest prevalence in the Chilean salmon industry, reaching mortality rates of up to 90% when an event occurs [2,3,4]. SRS incurs annual losses exceeding 600 million dollars, with 8 million dollars spent on antibiotic therapy. This increases the risk of antimicrobial resistance (AMR) and negatively impacts marine environments. Despite a decrease in antibiotic use in Chilean salmon farming, SRS still accounts for 90% of antimicrobials administered in salmon production [2,4,5,6,7,8]. Its facultative intracellular nature and biofilm formation confer low susceptibility to authorized antibiotics in Chilean aquaculture (florfenicol and oxytetracycline) [9,10,11], thereby reducing its exposure to these drugs and promoting chronic infections. The FAO has emphasized the urgent need to reduce antibiotic use and promote innovative alternatives for pathogen control in aquaculture [8,12].

Chitosan is a polymer obtained by deacetylation of chitin and has antimicrobial properties [13,14,15,16]. According to molecular weight and degree of deacetylation (DG) of chitosans and their chemical modifications, the performance of these biopolymers has been improved in these variables (minimum inhibitory concentration, polymer size, environmental pH, bacterial species) that restrict or modify their antibacterial activity [15,17,18,19,20,21]. Furthermore, this has allowed for significant changes and improvements in solubility and bioavailability for biomedical applications due to its properties of biocompatibility, biodegradability, and low toxicity [15,18,20,22,23,24,25,26,27]. This is the case with controlled chemical modifications to several sulfated chitosan polysaccharides, in which greater solubility and antibacterial efficacy, among other properties, have been reported [17,19,21,22,28,29,30,31].

In the present study, it was performed with sulfated chitosan (ChS), a polysaccharide with chemical modifications that mimic heparan sulfate (HS), a glycosaminoglycan (GAG) found in hosts that is used by various intracellular pathogens for cell adhesion and invasion [32,33] has been recently used to bind viruses and bacteria [26,33,34,35,36,37,38]. The high negative charge density of ChS [39] enables it to interact with bacterial surfaces, with both ionic and non-ionic attractions to negatively charged bacterial membranes, thereby promoting adhesion [22,23,24,28,40,41,42,43].

In this context, we suggest that this could lead to charge modification, membrane destabilization, and possible inhibition of adhesion or biofilm formation in *P. salmonis*. Previous studies reported by our research group have shown that ChS blocks viral and bacterial adhesion through mechanisms that mimic HS [34,35,36,39]. We developed ChS with low molecular weight, a determined degree of deacetylation, and controlled chemical substitution in its functionalization with sulfur. These particles have been patented as antimicrobial biomaterials for use in biomedical and biotechnological applications (US Patent 11,246,839 B2) [39]. Compared with ChC, these materials exhibit enhanced physicochemical properties and improved biological activity [39]. Currently, we have found no reports on the use of modified or chemically unmodified chitosan against *P. salmonis*.

Based on these properties, we hypothesized that ChS might interact with *P*. *salmonis* via ionic and non-ionic bonds, thereby acting as an HS analogue and impacting bacterial membrane integrity and the aggregation processes. This study aimed to evaluate the in vitro antibacterial activity of ChS against the *P. salmonis* LF-89 strain, including its effects on bacterial viability, membrane integrity, cellular morphology, and biofilm production. Understanding these interactions could inform the development of sustainable, biomaterial-based strategies for controlling SRS in aquaculture using a One Health approach.

## 2. Results

### 2.1. Elemental Characterization and Degree of Sulfation of Sulfated Chitosan (ChS)

**Figure 1:**
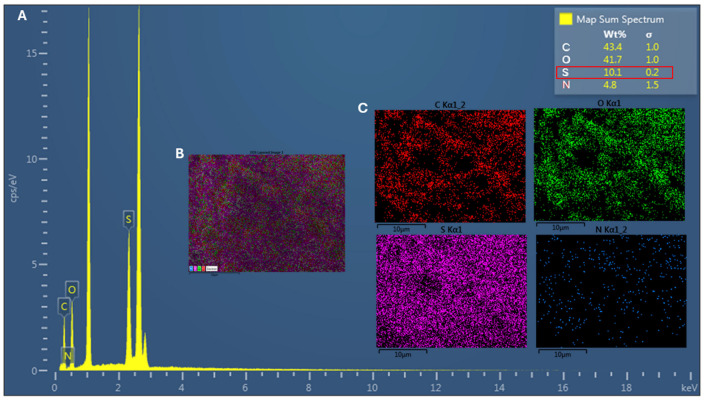
Energy-dispersive X-ray spectroscopy (EDS) analysis (**A**) and elemental map of sulfate chitosan sample inset (**B**). The left panel shows a representative EDS spectrum of the analyzed area, with characteristic peaks for C, O, S, and N. The inset table summarizes the semi-quantitative elemental composition (wt% distinguished in red box), indicating that C (43.4 wt%) and O (41.7 wt%) are the predominant elements, followed by S (10.1 wt%) and N (4.8 wt%). The right-hand panels present individual elemental distribution maps (**C**) for C (Kα1), O (Kα1), S (Kα1), and N (Kα1). Carbon and sulfur exhibit a relatively homogeneous distribution across the analyzed region, consistent with a carbon-rich sulfur-containing matrix. Oxygen shows a moderate and uniform signal, whereas nitrogen appears at a lower intensity and is more sparsely distributed.

Energy-dispersive X-ray spectroscopy (EDS) analysis revealed the presence of the main chemical elements expected for sulfated chitosan (ChS), namely carbon (C), oxygen (O), nitrogen (N), and sulfur (S) (Figure 1). The elemental composition of the analyzed sample was 43.4% C, 41.7% O, 10.1% S, and 4.8% N. These results support the successful chemical modification of the chitosan backbone. Notably, the detection of sulfur, absent in unmodified chitosan (ChC), provides clear evidence of sulfate group incorporation into the polymer structure.

Based on the sulfur-to-nitrogen (S/N) ratio obtained from EDS, the degree of sulfation (DS) was estimated to be approximately DS ≈ 0.92–0.95, indicating a high level of substitution of glucosamine units by sulfate groups. Elemental mapping further revealed a relatively homogeneous distribution of sulfur throughout the analyzed area, suggesting uniform chemical modification.

The introduction of sulfate groups is expected to increase the negative charge density of the polymer, thereby altering its physicochemical properties and potentially enhancing its interaction with bacterial cell surfaces. Overall, these results suggest extensive sulfation, likely involving a significant proportion of the available amino and hydroxyl groups in the glucosamine units.

### 2.2. Fourier Transform Infrared Spectroscopy (FTIR) of ChS

**Figure 2:**
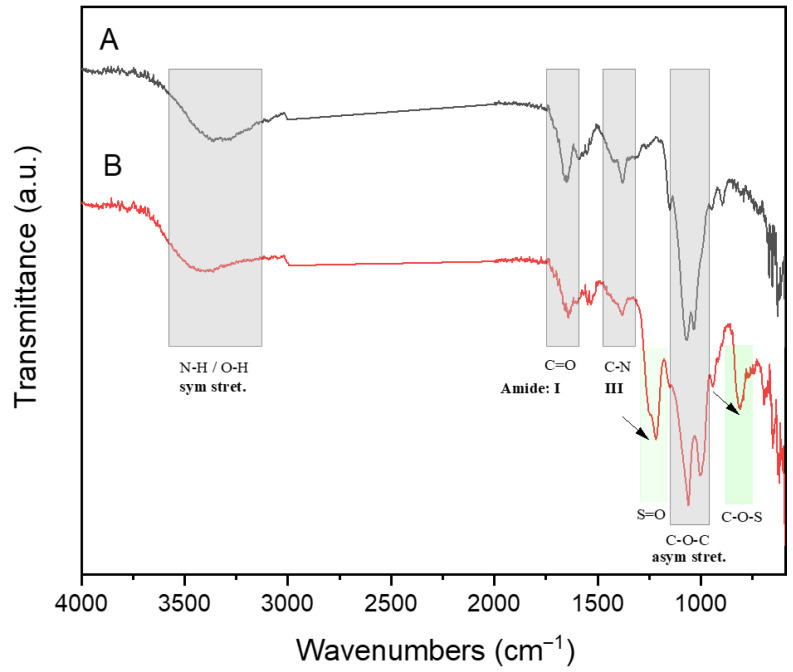
FTIR spectra of ChC (A) and ChS (B) derivatives. Light gray and green shaded bars indicate the characteristic absorption bands of commercial chitosan and sulfated groups, respectively. Black arrows highlight two new absorption bands at 1216 cm^−1^ and 808 cm^−1^, attributed to S=O and C-O-S bonds, respectively, in ChS, confirming the presence of sulfation groups.

Figure 2 shows the FTIR spectra of the ChC (A) and ChS (B) derivatives, which show the characteristic absorption bands of commercial chitosan (Figure 2A) and sulfated groups (Figure 2B), respectively. In general, FT-IR analysis was used to evaluate the incorporation of the sulfate group into the ChC polymer. Figure 2A shows the characteristic absorption bands of commercial ChC corresponding to symmetric stretching of N-H and O-H at about 3300 cm^−1^, and the absorption bands of I and III amide groups ascribed to C=O and C-N bonds, respectively, and the asymmetric stretching of C-O-C bonds of the ChC structure polymer [44]. However, Figure 2B of ChS highlights two new absorption bands at 1216 cm^−1^ and 808 cm^−1^, attributed to S=O and C-O-S bonds, respectively, which provide clear evidence that the sulfate groups are successfully incorporated into ChC; similar results have also been reported elsewhere [45,46].

### 2.3. X-Ray Diffraction (XRD) Analysis of ChS

**Figure 3:**
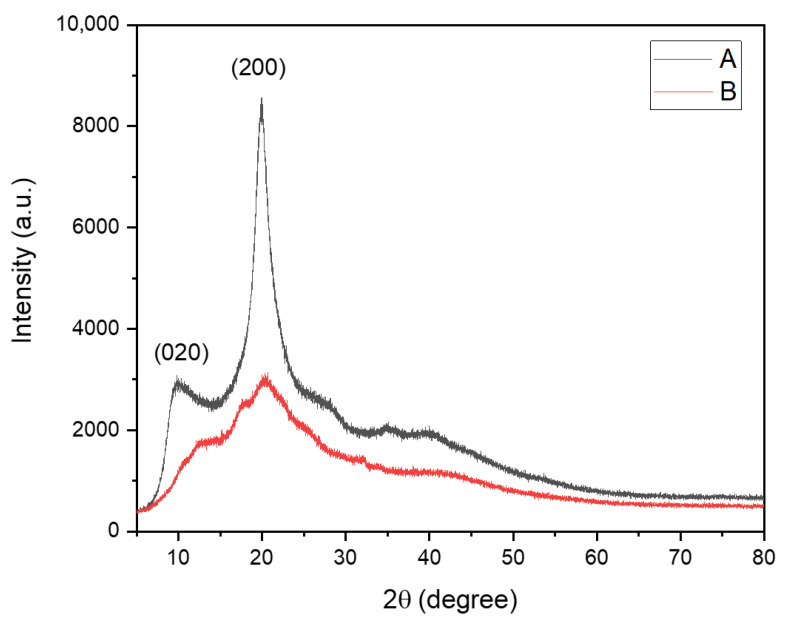
X-ray diffraction (XRD) patterns of commercial chitosan (ChC) (A) and sulfated chitosan (ChS) (B). Colored curves are included for clarity. The ChC diffractogram (A) shows two characteristic reflections at 2θ ≈ 10° and 2θ ≈ 20°, corresponding to the (020) and (200) crystallographic planes, respectively, typical of the semi-crystalline structure of chitosan [47]. In contrast, the ChS pattern (B) exhibits reduced peak intensity and broadening in the 10–25° 2θ range, indicating decreased crystallinity and the formation of a more amorphous structure after sulfation.

Figure 3 shows the X-ray diffraction (XRD) patterns of commercial chitosan (ChC) (A) and sulfated chitosan (ChS) (B). As observed in Figure 3A, ChC exhibits two well-defined diffraction peaks at 2θ ≈ 10° and a more intense peak at 2θ ≈ 20°, corresponding to the characteristic semi-crystalline structure of chitosan, typically associated with the (020) and (200) crystallographic planes, respectively, as reported in the literature [47].

In contrast, the XRD pattern of ChS (Figure 3B) shows a pronounced decrease in peak intensity and significant broadening of the diffraction profile, particularly in the region between 10° and 25° 2θ. The sharp peak at ~20° observed in ChC is replaced by a broad, low-intensity halo, indicating a loss of long-range crystalline order. This change reflects the disruption of the intermolecular hydrogen-bonding network due to the introduction of sulfate groups.

Additionally, the ChS diffractogram displays a more diffuse scattering pattern across the entire range, suggesting the formation of a predominantly amorphous structure. No new well-defined crystalline peaks are observed, indicating that sulfation does not generate new ordered domains but rather reduces structural organization.

Quantitative analysis of crystallinity in the range of 2θ = 5–25° revealed a marked decrease in the degree of crystallinity (%Crystallinity), from 20.6% in ChC to 6.7% in ChS. This substantial reduction confirms that sulfation induces a transition from a semi-crystalline to a predominantly amorphous structure.

### 2.4. Antibacterial Activity

**Figure 4:**
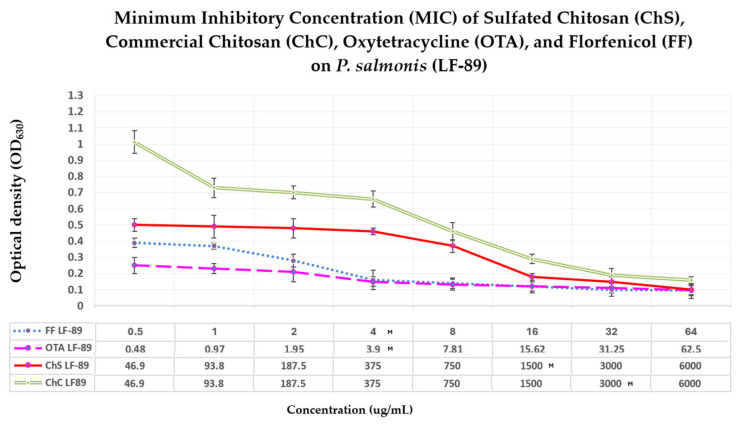
The minimum inhibitory concentration (MIC) of chitosan derivatives and antibiotics against *P. salmonis* LF-89. The figure shows the MIC values obtained for ChS, ChC, OTA, and FF. The letter M indicates the MIC of each active ingredient. Optical density measurements were used to determine bacterial growth inhibition. ChS exhibited a MIC of 1500 µg/mL, whereas ChC required 3000 µg/mL to inhibit bacterial growth. In contrast, OTA and FF showed lower MIC values (3.9 µg/mL and 4 µg/mL, respectively), indicating higher antibacterial potency. The results also demonstrate a concentration-dependent inhibitory effect of chitosan derivatives on *P. salmonis*.

The antibacterial activity of sulfated chitosan (ChS) and commercial chitosan (ChC) against *P. salmonis* LF-89 was evaluated by determining their minimum inhibitory concentration (MIC) and minimum bactericidal concentration (MBC). The results are summarized in Figure 4 and Table 1. ChS exhibited a MIC of 1500 µg/mL, whereas ChC required 3000 µg/mL to inhibit bacterial growth, indicating that sulfation approximately doubled the antibacterial activity of chitosan against *P. salmonis*.

In contrast, antibiotics currently authorized for use in Chilean aquaculture showed substantially higher antibacterial potency. Oxytetracycline (OTA) and florfenicol (FF) exhibited MIC values of 3.9 µg/mL and 4.0 µg/mL, respectively, corresponding to approximately 385- and 375-fold higher potency than ChS.

Despite its lower intrinsic potency compared to conventional antibiotics, ChS exhibited a clear concentration-dependent inhibitory effect on *P. salmonis* over the 144 h incubation period. Notably, comparing ChS and ChC indicates that sulfation substantially enhances the biopolymer’s antibacterial activity.

#### Bacterial Viability and Bacterial Membrane Permeability

**Figure 5:**
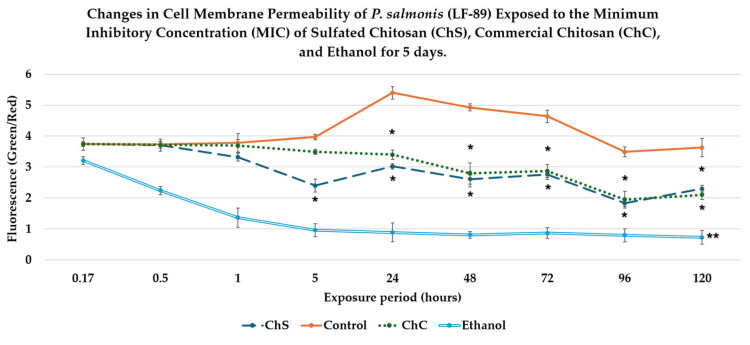
Changes in bacterial membrane permeability of *P. salmonis* are shown following exposure to ChS, ChC, and ethanol for 5 days (120 h of incubation), compared with the inoculum control. Cell membrane permeability was assessed using LIVE/DEAD fluorescence staining based on the ratio between green fluorescence (SYTO9, viable cells) and red fluorescence (propidium iodide, membrane-damaged cells). The decrease in the Green/Red ratio indicated greater intracellular propidium iodide (PI) uptake and, therefore, greater alteration of the bacterial cell membrane. Cultures were exposed to MIC of ChS (1500 µg/mL) and ChC (3000 µg/mL). Ethanol (50%) was used as a positive control for membrane disruption. The data represent the mean fluorescence ratio obtained from three independent replicates over a 5-day period. Asterisks (*) indicate statistically significant relative to the untreated control (*p* < 0.05), whereas double asterisks (**) indicate stronger significance (*p* < 0.01) of ethanol after 30 min.

Bacterial membrane permeability after exposure to chitosan derivatives was evaluated using LIVE/DEAD fluorescence staining, which quantifies the ratio of green fluorescence (SYTO9, intact cell membranes) to red fluorescence (propidium iodide, damaged membranes). A higher Green/Red fluorescence ratio indicates a normal cell membrane structure and function. In contrast, lower values reflect increased membrane permeabilization and can be used to assess decreases in viability, as observed in other studies (e.g., SEM in bacteria and inoculation into culture media to demonstrate the absence of bacterial growth). During the early exposure phase (10–30 min), no statistically significant differences (*p* > 0.05) were detected between the chitosan treatments and the untreated control (Figure 5). By contrast, ethanol used as a positive control for membrane disruption produced a rapid and significant reduction in bacterial viability within the first 10 min of exposure (*p* < 0.05).

After 1 h of exposure, both biopolymers exhibited a slight decrease in the green/red fluorescence ratio compared with the control. However, these differences were not statistically significant. ChS was tested at its MIC of 1500 µg/mL, whereas ChC required a higher concentration of 3000 µg/mL.

Clear treatment-dependent differences became evident during the intermediate exposure period (5–48 h). At 5 h, the control inoculum maintained stable membrane permeability values, whereas ChS significantly reduced the Green/Red fluorescence ratio compared with both the control and the ChC treatment (*p* < 0.05). Between 24 h and 48 h, both chitosan derivatives induced a significant reduction in the Green/Red fluorescence ratio relative to the control (*p* < 0.05).

Despite differences in MIC values, no statistically significant differences were observed between ChS and ChC during this period, suggesting comparable effects once the polymers reached their effective concentrations. During prolonged exposure (72–120 h), the untreated control maintained consistently high fluorescence ratios, indicating stable membrane permeability in the absence of treatment. By contrast, both chitosan derivatives were found to produce a sustained reduction in the Green/Red fluorescence ratio (*p* < 0.05), confirming a time-dependent effect. Also, the *P. salmonis* cells cultured in Austral-SRS medium for 14 days to evaluate bacterial recovery showed no detectable growth after 120 h of exposure to the treatments, indicating a time-dependent lethal effect.

Overall, the Green/Red fluorescence ratio assay indicates that exposure to chitosan polymers results in a progressive increase in bacterial membrane permeability of *P. salmonis*. Notably, ChS produced effects comparable to those of ChC when applied at half the concentration (1500 µg/mL versus 3000 µg/mL), supporting the idea that polymer sulfation enhances antibacterial efficiency. Ethanol served as an effective positive control, inducing a rapid and sustained increase in bacterial membrane alterations.

### 2.5. Fluorescence Microscopy Analysis

**Figure 6:**
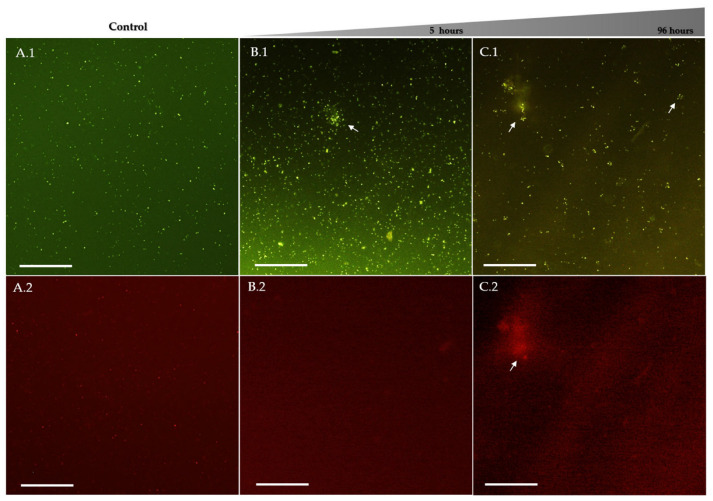
Fluorescence microscopy of *P. salmonis* (×10) after exposure to commercial chitosan (ChC). Fluorescence microscopy of *P. salmonis* (LF-89) stained with LIVE/DEAD dyes (SYTO9: green, intact membranes; PI: red, membrane-damaged cells). Panels (**A**–**C**) correspond to untreated control (**A**), 5 h (**B**), and 96 h (**C**) exposure to ChC at MIC (3000 µg/mL). Panels .1 and .2 show SYTO9 and PI channels, respectively. The untreated control exhibits predominantly green fluorescence, indicating intact membranes and dispersed cells. After 5 h of ChC exposure, small bacterial aggregates (arrows) are observed, with green fluorescence still predominant. At 96 h, aggregates become more frequent (arrows), with increased but diffuse red fluorescence, suggesting partial membrane permeabilization. Scale bar = 100 µm.

Fluorescence microscopy with LIVE/DEAD staining was used to assess the integrity of the membrane in *P. salmonis* after exposure to chitosan derivatives. This staining method uses two fluorophores: SYTO9, which penetrates all cells and emits green fluorescence, and propidium iodide (PI), which only penetrates cells with compromised membranes and emits red fluorescence. When both dyes are intracellular, PI displaces SYTO9, resulting in predominant red fluorescence, indicating membrane damage. In the untreated control group, bacterial cells displayed predominantly green fluorescence with minimal red signal (Figure 6A). The cells were mostly isolated and distributed homogeneously, with no evident bacterial microaggregates, indicating preserved membrane integrity during the experiment.

Exposure to 50% ethanol, used as a positive control for membrane disruption, resulted in a rapid increase in red fluorescence, accompanied by a reduction in green fluorescence. This result is consistent with the known mechanism of ethanol in Gram-negative bacteria, which involves disruption of the bacterial membrane. In cultures exposed to ChC, both green and red fluorescence signals were detected (Figure 6B,C). Small bacterial aggregates were observed after 5 h of exposure, with green fluorescence predominating and limited red fluorescence. After prolonged exposure (96 h), aggregates became more frequent but did not show substantial increases in size. Red fluorescence remained relatively diffuse and less intense within these aggregates, suggesting partial membrane permeabilization.

**Figure 7:**
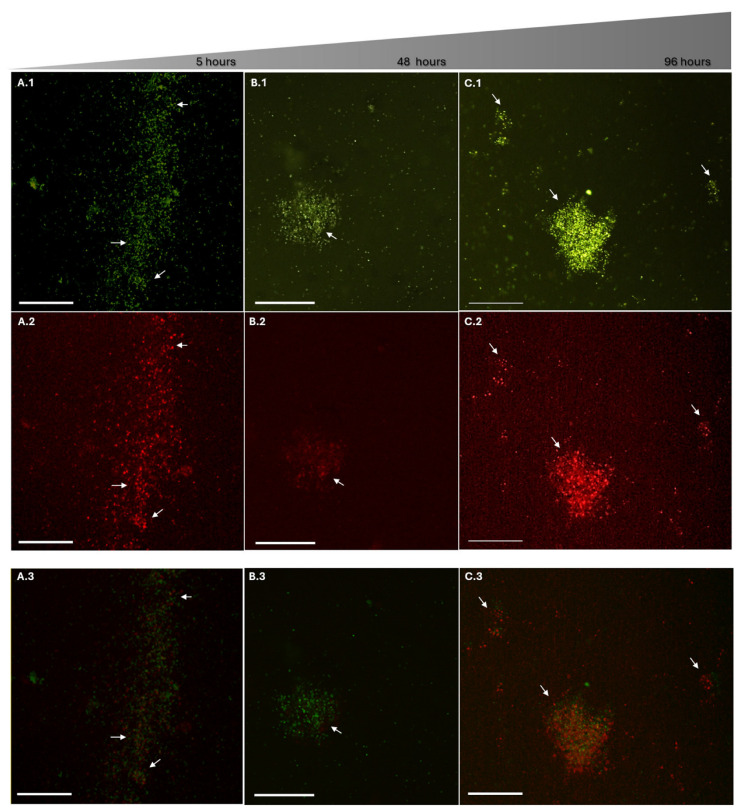
Fluorescence microscopy of *P. salmonis* (×10) after exposure to sulfated chitosan (ChS). Fluorescence microscopy of *P. salmonis* (LF-89) after exposure to ChS at its MIC (1500 µg/mL), using LIVE/DEAD staining (SYTO9: green fluorescence, intact membranes; propidium iodide (PI): red fluorescence, membrane-damaged cells). Panels (**A**–**C**) correspond to exposure times of 5 h (**A**), 48 h (**B**), and 96 h (**C**). Panels (**A.1**–**C.2**) show the individual fluorescence channels (SYTO9 and PI), while panels (**A.3**–**C.3**) represent merged images illustrating the spatial overlap of green and red signals. At early exposure times (5–48 h), green fluorescence predominates, indicating largely intact membranes, with the formation of small bacterial aggregates (arrows). At 96 h, larger and more frequent aggregates are observed, with increased red fluorescence and evident green/red overlap (yellow–orange regions), consistent with progressive membrane damage and increased PI permeability. A reduced number of green-only cells is observed a later time points, suggesting decreased bacterial viability. Scale bar = 100 µm.

In contrast, cultures exposed to ChS exhibited more pronounced alterations in fluorescence patterns (Figure 7). As early as five-hour of exposure, bacterial microaggregates were already evident, predominantly showing green fluorescence with limited red signal. However, between 24 h and 48 h, a marked increase in red fluorescence within these aggregates was observed, indicating progressive membrane permeabilization. During prolonged exposure (72–120 h), aggregates became larger and more frequent, with red fluorescence becoming comparable to or exceeding green fluorescence. Additionally, satellite or peripheral aggregates appeared surrounding the main clusters, a feature not observed at earlier time points, suggesting extensive membrane damage and increased propidium iodide (PI) penetration.

These qualitative observations are consistent with quantitative fluorescence measurements, which showed a progressive decrease in the Green/Red fluorescence ratio following ChS exposure. Merged images (Figure 7(A.3–C.3)) obtained by overlaying the SYTO9 (green) and PI (red) channels further illustrate this transition. At 5 and 48 h, green fluorescence predominated. In contrast, at 96 h, a substantial increase in red fluorescence was observed, along with regions of signal overlap (yellow–orange), indicating mixed populations with varying degrees of membrane integrity.

Notably, the merged image at 96 h (Figure 7(C.3)) reveals a central aggregate with strong red fluorescence, surrounded by areas of green/red colocalization, suggesting intermediate physiological states associated with progressive membrane disruption. This pattern indicates a gradual transition rather than an abrupt shift between viable and non-viable cells.

**Figure 8:**
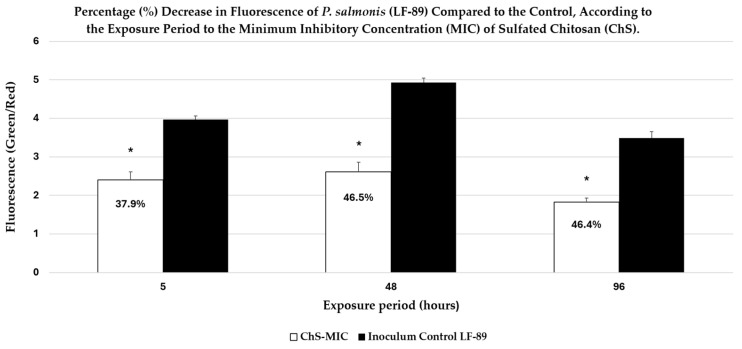
Decrease in the Green/Red ratio of *P. salmonis* after exposure to ChS-MIC. Bacterial membrane permeability was assessed using LIVE/DEAD staining and expressed as the Green/Red fluorescence ratio. Cultures were exposed to ChS at its MIC (1500 µg/mL), and data are shown for 5, 48, and 96 h. ChS reduced (*p* < 0.05) the Green/Red ratio compared with the untreated control * (*p* < 0.05). The decrease in the Green/Red ratio indicated greater intracellular PI uptake and, therefore, greater alteration of the bacterial cell membrane.

These observations are supported by quantitative fluorescence analysis (Figure 8), which shows a significant decrease in the Green/Red fluorescence ratio after exposure to ChS. As shown in Figure 8, exposure to ChS reduced the Green/Red ratio by 37.9%, 46.5%, and 46.4% at 5, 48, and 96 h, respectively, compared with the untreated control.

### 2.6. Scanning Electron Microscopy (SEM) Analysis

**Figure 9:**
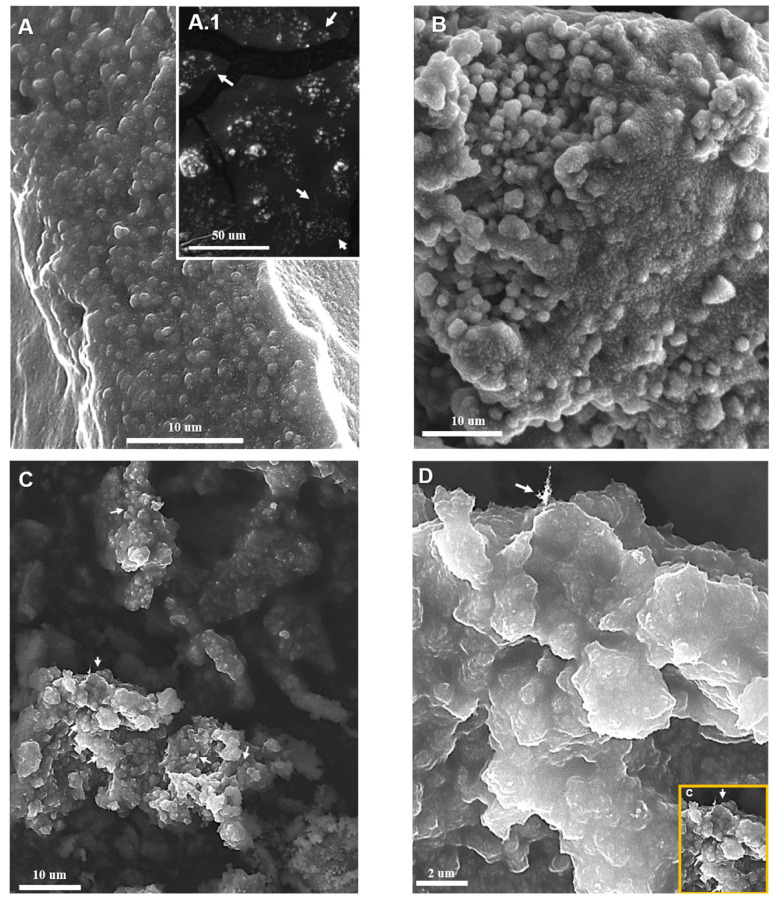
Scanning electron microscopy of *P. salmonis* was exposed to chitosan derivatives. Morphological comparison of *P. salmonis* (LF-89) cultures after 24 h exposure to the minimum inhibitory concentrations of commercial chitosan (ChC) and sulfated chitosan (ChS). (**A**) Micrograph (×2500): Untreated control showing spherical and coccoid bacteria with smooth surfaces, well-defined cell boundaries, and intercellular demarcation is distinguishable (arrow). (**A.1**) Micrograph (×300): Image of the inoculum control at lower magnification showing more dispersed bacteria (arrows) and with little bacterial aggregate formation. (**B**) Micrograph (×1900): Cultures exposed to ChC (3000 µg/mL), showing moderate surface roughness, bacterial aggregation, and reduced preservation of coccoid shape. (**C**) Micrograph (×1500): Cultures exposed to ChS (1500 µg/mL), showing large compact aggregates, loss of cellular contours (arrows), and intercellular demarcation is not distinguishable. (**D**) Magnification of micrograph (**C**) (×6500): Surface irregularities and severe distortion of the bacterial envelope (arrow), without distinction of intercellular boundaries.

Scanning electron microscopy (SEM) was used to examine morphological alterations in *P. salmonis* cultures exposed to the MICs of ChS and ChC for 24 h (Figure 9). Control cultures of *P. salmonis* without chitosan polymer derivatives exposure displayed spherical to coccoid bacterial cells with relatively smooth surfaces and well-defined cellular boundaries (Figure 9(A,A.1)). The bacteria were frequently observed as isolated cells and occasionally forming small microclusters. The cell morphology appeared intact, with no visible surface depressions or structural alterations. Cultures exposed to the MIC of ChC showed moderate morphological changes compared to the control (Figure 9B). Although most bacterial cells retained their spherical morphology, surface roughness and slight deformation of the coccoid shape were evident. The cells frequently appeared clustered and partially embedded within a matrix-like structure. However, the cell boundaries remained distinguishable in most areas.

In contrast, cultures exposed to the ChS MIC exhibited significantly more pronounced morphological alterations (Figure 9C,D). Large irregular aggregates composed of densely packed bacterial cells were observed, accompanied by extensive cell coalescence and loss of clear intercellular boundaries. At higher magnification, cells showed surface depressions, reduced intracellular volume, and severe distortion of the outer cell envelope. The formation of compact aggregates and the loss of normal coccoid morphology suggest strong interactions between bacterial cells and the sulfated chitosan polymer. These structural alterations were more extensive and frequent than those observed in cultures treated with ChC. Overall, SEM analysis indicates that exposure to ChS induces substantial structural damage in *P. salmonis*, consistent with membrane disruption observed by fluorescence microscopy and with the reduction in the Green/Red ratio (Increased intracellular PI uptake) in fluorescence assays. Also, the *P. salmonis* cells cultured in Austral-SRS medium to evaluate bacterial recovery showed no detectable growth after 120 h of incubation under the treatments. This finding, in the case of ChS, complements the results of the Green/Red ratio studies and SEM images. It is consistent with a possible mechanism of action of ChS based on disruption of the cell membrane, which could irreversibly compromise bacterial viability.

### 2.7. Evaluation of Biofilm Production

**Figure 10:**
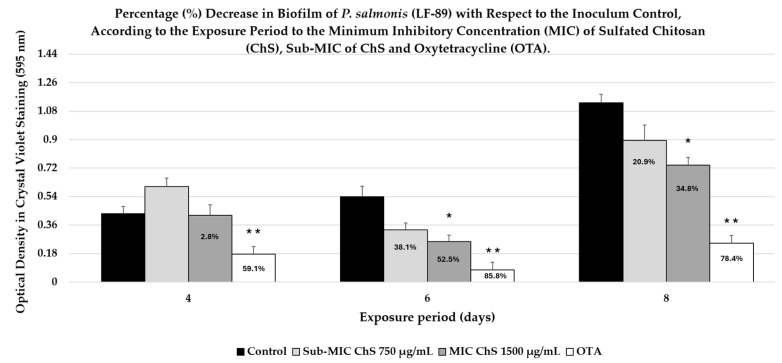
Percentage reduction in biofilm in *P. salmonis* cultures exposed to ChS MIC (1500 µg/mL), Sub-MIC (750 µg/mL), and OTA (5 µg/mL) as a positive control, compared to the untreated control, according to the number of days of experimental incubation. A reduction (*p* < 0.05) in biofilm was observed when the bacteria were exposed to ChS MIC on days 6 and 8 of incubation compared to the inoculum control. The Crystal Violet staining method allowed identification of statistically significant differences (* *p* < 0.05 and ** *p* < 0.01) between treatments based on corrected optical density (OD) readings.

Biofilm formation was evaluated during the 4 days of incubation. The untreated bacterial control exhibited a mean OD of 0.433 ± 0.044, indicating initial biofilm establishment under the experimental conditions (Figure 10). According to the Stepanović classification method, the strain was categorized as a weak biofilm producer. This suggests that, at this early stage, biofilm formation is not yet fully developed. The antibiotic control exhibited markedly lower biofilm (0.177 ± 0.019), corresponding to a 59.1% reduction (*p* < 0.05) relative to the untreated control. Treatment with ChS at 750 µg/mL increased biofilm formation (0.604 ± 0.053), suggesting a slight stimulation relative to the control. In contrast, ChS at 1500 µg/mL yielded an OD of 0.421 ± 0.067, representing a minimal 2.8% reduction (*p* > 0.05) and indicating a negligible inhibitory effect at this time point. Conversely, the OTA-treated group (the antibiotic control) showed a significant reduction in biofilm formation compared to all other groups (*p* < 0.001).

During the 6-day incubation period, according to Stepanović’s classification method, the biofilm production by the strain at 6 days was classified as weak. However, the amount of biofilm quantified after 6 days (OD = 0.539 ± 0.068) increased compared to 4 days of incubation (see Figure 8). In this context, the untreated bacterial control exhibited the greatest biofilm biomass compared to the other groups. Conversely, treatment with ChS resulted in a dose-dependent reduction in biofilm formation. Specifically, ChS at 750 µg/mL reduced biofilm formation by 38.6%. This decrease was not significant (*p* > 0.05) compared with the control. However, increasing the concentration to 1500 µg/mL resulted in a 52.5% reduction (*p* < 0.05), indicating improved antibiofilm activity from exposure to the MIC of ChS. The most pronounced effect was observed with the positive control (OTA, 5 µg/mL), resulting in an 85.9% reduction in biofilm biomass (*p* < 0.001), confirming both the assay’s sensitivity and the validity of the experimental design.

After 8 days of incubation, the bacterial control showed an average optical density (OD) of 1.133 ± 0.053, indicating an increase in adhered biofilm biomass compared to previous experiments (Figure 8). According to Stepanović’s classification, the strain was classified as a moderate biofilm producer, consistent with the high OD values observed in the control group during this experimental period. The evaluation of the treatments revealed a differential reduction in biofilm formation. ChS at 750 µg/mL showed an OD of 0.896 ± 0.096, corresponding to a 20.9% decrease compared to the control. However, this effect was not statistically significant (*p* > 0.05), suggesting that although there is an inhibitory trend, the experimental variability in this group may have prevented detection of statistical significance; a biological decrease was evident.

In contrast, the MIC of ChS (1500 µg/mL) resulted in an OD of 0.739 ± 0.049, corresponding to a 34.8% reduction, which was statistically significant (*p* < 0.05). This finding demonstrates a dose-dependent effect, indicating that increasing the ChS concentration allows it to exceed the threshold necessary to significantly interfere with biofilm maturation or production. For its part, the antibiotic control treatment exhibited the greatest inhibitory efficacy during this experimental period, with an OD of 0.245 ± 0.04, corresponding to a 78.4% decrease (*p* < 0.01) compared to the control. This result validates the model using positive control, which remained constant throughout the three experimental periods, demonstrating superior inhibitory capacity regardless of the biofilm’s maturation stage.

## 3. Discussion

To contextualize the observed results and establish a mechanistic framework, the following discussion integrates the structural and functional properties of sulfated chitosan (ChS) with antibiotic use and *P. salmonis* biology. The behavior observed for sulfated chitosan (ChS) may differ from that of unmodified chitosan due to its structural biomimicry with heparan sulfate (HS), its net negative charge resulting from sulfur functionalization, and its low molecular weight. This is particularly relevant in *P. salmonis*, a facultative intracellular Gram-negative pathogen with reduced susceptibility to conventional antibiotics such as florfenicol (FF) and oxytetracycline (OTA), especially under biofilm-associated or chronic infection conditions [48,49,50,51].

The successful incorporation of sulfate groups into the chitosan backbone was supported by elemental characterization and previously established deacetylation levels [39], yielding a high degree of sulfation (DS ≈ 0.92–0.95) in the present study. However, it should be noted that EDS provides only semi-quantitative information; therefore, DS values should be interpreted as indicative rather than absolute. Overall, these results suggest extensive sulfation, likely involving a substantial proportion of the available amino and hydroxyl groups in the glucosamine units.

FTIR analysis further confirmed the presence of sulfate groups after chemical modification, in agreement with previous reports [45,46]. This level of substitution is consistent with sulfated chitosan derivatives that retain structural stability while exhibiting enhanced biological activity [22]. In addition, XRD analysis revealed a marked reduction in crystallinity in ChS compared to ChC, without evidence of new crystalline phases [47], indicating a transition toward a more amorphous structure. This structural change is likely associated with increased polymer chain mobility and greater accessibility of functional groups, facilitating enhanced interactions with bacterial surfaces and biomolecules, including proteins involved in cell adhesion [52,53].

The MIC results indicated a concentration-dependent effect of ChS on *P. salmonis* in vitro. While the licensed antibiotics for use in aquaculture (FF and OTA) have lower MIC than chitosans (ChS and ChC). Unmodified chitosans generally exert their action through non-specific mechanisms on the cell membrane or other bacterial structures [17,21]. ChC usually requires higher MICs than licensed antibiotics, and chemical modifications are necessary to improve its potency, efficacy, or bioavailability, as reported for polymer-based antimicrobial agents [17,54,55]. ChS showed a 50% reduction in MIC compared to ChC, suggesting that the chemical surface modification increases its interaction with the bacterial surface. These results indicate that, although chitosan required higher concentrations to inhibit bacterial growth, it has a progressive antibacterial effect, a low potential for resistance development, low toxicity, and minimal environmental impact [20,21,22,23,25].

The combined results from membrane integrity analysis (qualitative and quantitative) using fluorescence microscopy, together with morpho-structural observations obtained by SEM, demonstrated distinct antibacterial behaviors between ChS and ChC. The predominance of red fluorescence and the presence of yellow–orange overlapping signals indicate a progressive loss of membrane integrity, consistent with membrane disruption mechanisms. Furthermore, the time-dependent decrease in the Green/Red ratio observed between 72 h and 96 h of exposure suggests that sulfate groups enhance chitosan’s antimicrobial activity by promoting sustained membrane permeabilization. The relationship between increased PI uptake and irreversible ultrastructural damage supports a mechanism driven by progressive membrane disruption, as described in other microorganisms exposed to membrane-targeting agents [56,57,58].

It is also important to distinguish the formation of bacterial aggregates, which increased in size and frequency with exposure time to ChS. Similar aggregation phenomena have been described for modified chitosans and are often attributed to polymer-mediated crosslinking between bacterial cells. In this process, aggregation is coupled with membrane disruption, leading to leakage of intracellular components and antimicrobial effects [54,59,60].

Notably, reports on *P. salmonis* exposure to modified or unmodified chitosan are absent or scarce. However, regarding this aspect, some researchers [61] discuss the findings of other authors [62,63,64] that unmodified, high-molecular-weight polycationic chitosans can form a dense polymer film on the cell surface. This blocks nutrient exchange by coating the outer membrane porins of Gram-negative bacteria, leading to bacterial death [64]. It was also reported here that flocculation can be detected by SEM, which shows vesicular structures in the outer membranes of *E. coli* and *S. typhimurium* treated with chitosan [62].

Other studies [57] described the antibacterial activity of ferulic acid-grafted chitosan derivatives against *L. monocytogenes*, *P. aeruginosa*, and *S. aureus*. Fluorescence staining with PI demonstrated increased membrane permeability and showed that the cells were found in aggregates. Meanwhile, SEM observations revealed profound changes in cell morphology, with cells clustering to form aggregates and a marked increase in cell size compared to their untreated controls. The authors proposed that adsorption of chitosan derivatives onto bacterial membranes could alter cell–cell interactions and, in turn, contribute to localized cellular aggregation during the antimicrobial process.

In contrast, other reports on the antibacterial activity of films with modified polycationic chitosans (graphene oxide, among other derivatives) were evaluated in *Campylobacter jejuni* and *Listeria monocytogenes*, where increased PI uptake indicates membrane permeabilization. SEM images revealed cell wall disruption and leakage of intracellular components, consistent with the classic mechanism of electrostatic interactions between chitosan and bacterial surfaces. However, they did not describe the phenomenon of bacterial aggregation [58].

Other authors exposed *S. aureus*, *P. aeruginosa,* and *E. coli* to silver nanoparticles combined with chitosan. They demonstrated that bacterial aggregates formed in all species with these polycationic nanocomposites at the highest tested chitosan concentration. Aggregates were more evident and frequent in *E. coli* than in the other species [59]. In the current work, the formation of large aggregates observed in SEM and fluorescence images suggests that ChS may promote the formation of polymer-bacteria complexes and may be associated with antibacterial activity. However, the formation of cell aggregates in bacteria exposed to chitosan, with or without modifications, is a variable phenomenon whose complexity remains to be further investigated.

Although some of the previously cited authors associate bacterial aggregation with the antibacterial effects of exposure to these polymers, Laanoja et al. [59] argue that greater importance should be placed on studying bacterial cell aggregates. These reports describe how interactions between bacteria and harmful nanoparticles can disrupt the cell membrane, leading to leakage of intracellular contents and modulation of cell surface properties. This could trigger the production of extracellular polymeric substances and promote bacterial aggregation as a defensive response [65].

In this regard, previous studies [48] have demonstrated that *P. salmonis* can form structured cellular aggregates under nutrient-limited conditions (48 h), in contrast to bacteria cultured in nutrient-rich conditions. Also, enzymatic treatment with cellulose led to marked disaggregation of these structures, suggesting that they were stabilized by an extracellular polymeric substance (EPS) matrix, a characteristic feature of biofilms. SEM further revealed that these aggregates corresponded to dense, three-dimensional structures in which bacterial cells were embedded within a rigid extracellular matrix. The authors proposed that this EPS-mediated aggregation acts as a protective strategy, facilitating environmental persistence and host colonization.

In contrast, the microaggregates observed following exposure to both ChS and ChC do not display features consistent with mature EPS-embedded biofilms. Instead, SEM analysis revealed irregular clustering of bacterial cells (with surface morphological changes) lacking a continuous extracellular matrix, suggesting a distinct aggregation mechanism. Given that these experiments were conducted in nutrient-rich SRS medium supplemented, conditions that do not typically induce EPS-driven aggregation, after 48 h of incubation and shaking. The observed clustering is more likely associated with physicochemical interactions induced by the polymers rather than a canonical biofilm developmental process.

Notably, aggregation was more pronounced in the presence of ChS, indicating that polymer-induced cell clustering may occur independently of the classical electrostatic interactions described for chitosan without modification. This suggests that alternative mechanisms, such as polymer bridging, surface adsorption, or modulation of intercellular forces, may contribute to bacterial aggregation under these conditions.

This work provides evidence of bacterial aggregates occurring with a sulfur-modified chitosan, which has a net negative charge. Therefore, the formation of bacteria-ChS complexes between this polymer and *P. salmonis* cannot be explained exclusively by electrostatic or polar attractions, as is the case with polycationic chitosans. However, bacterial aggregation could occur differently with modified chitosans, depending on the derivative type or functionalization [17,21,24].

Previous studies evaluating sulfated chitosan (ChS) with low degrees of sulfation have reported moderate antibacterial activity, with broad-spectrum activity against *E. coli* and *S. aureus*. Importantly, antimicrobial efficacy increased proportionally with the degree of sulfation (DS) [66]. Higher levels of sulfation increase the polymer’s negative charge density, which appears to improve its interaction with bacterial surfaces. For instance, derivatives with a DS of approximately 86% exhibited significantly enhanced antibacterial activity, particularly against *E. coli*, compared to *S. aureus*, suggesting a greater effectiveness against Gram-negative bacteria [67].

In this sense, the increased antibacterial activity of ChS [68] supports the results of the present research. Combined with other reported variables, such as polymer concentration and size, ambient pH, Gram-negative bacterial species [15,17,18,21], and, probably, the bacterial exposure period to the biopolymer, this suggests a different pattern of antibacterial activity to ChC.

Accordingly, one explanation for the biomimetic properties of sulfur-modified polymers is the presence of residual amino groups in their structure, which may confer polyampholytic characteristics similar to those of some sulfated glycosaminoglycans (GAGs), such as HS. This allows them to interact with a wide variety of GAG-binding proteins in the extracellular matrix and mediate various functions, such as adhesion [69], antimicrobial activity [26,67], and effects of intermolecular bonding, protein aggregation, or coagulation [70,71,72]. In this regard, some authors have described how chitosan derivatives retain some of these biological properties and exhibit antibacterial activity [22,28,29,30,31].

These properties are fundamental, as they suggest that the net negative charge of ChS, like the polyanionic structure of GAGs such as HS [26,28], likely interacts with Gram-negative bacteria, including *P. salmonis*, without significant restrictions [33,37,38,73]. In this sense, this could facilitate, through previously described mechanisms of ionic and non-ionic interactions [22,28], the binding or adhesion between ChS and *P. salmonis*.

Other authors have reported that the mechanical, chemical, or biological characteristics of chitosans, such as aggregation, molecular agglutination, blood coagulation, or contaminant binding [74,75,76,77], are modified or improved depending on their conjugations with other molecules [78,79,80]. Therefore, the properties of sulfur-containing chitosans mimicking HS, such as bacterial adhesion, protein aggregation, coagulation, or the formation of intermolecular complexes with enzymes/proteins [21,22,23,24,26,28,81,82,83], may also likely contribute to the alteration of the bacterial membrane by modifying its permeability, given that *P. salmonis* shares conserved structures with Gram-negative bacteria, such as the presence of an outer membrane with lipoproteins, polysaccharides and porins, which likely interacted with ChS as a HS biomimetic [39], producing a comparable effect of protein aggregation, agglutination, or coagulation, through the possible formation of supramolecular complexes between ChS and molecules on the bacterial surface, causing subsequent disruption of membrane integrity.

In this context, chemical modification of chitosan is proposed as a strategy to enhance its biological activity and expand its range of applications. Sulfate groups in chitosan introduce negative charges on the polymer backbone, altering its electrostatic properties and enabling interactions similar to those of GAGs, such as HS [21,22,23,26,28,81,82]. Sulfated polysaccharides have been extensively investigated due to their antiviral, antibacterial, and anti-adhesion properties, which are often attributed to their ability to interfere with host–pathogen interactions [23,26,34,35,36,39].

The results of this study provide information on the antibacterial activity of sulfur-modified chitosans, distinguishing their effect or affinity for the outer membrane of *P. salmonis*. It is worth noting that in intracellular Gram-negative bacteria such as *P. salmonis*, LPS differs from that of other Gram-negative bacteria, possibly due to lower surface exposure or structural differences in the core or the O-antigen oligosaccharide, which distinguishes it from other Gram-negative bacteria [84]. Its interactions with sulfated or non-sulfated chitosans may differ or be more complex. That is why it is necessary to delve deeper into studies of possible ionic and non-ionic interactions between the cell membrane of *P. salmonis* and negatively charged sulfated polymers.

In this context, interactions between ChS and the outer membrane of *P. salmonis* could also interfere with the surface structures that mediate adhesion in this bacterium, such as outer membrane vesicles (OMVs), which facilitate the secretion of virulence factors and participate in interbacterial communication and biofilm formation [85,86,87]. In this regard, type IV pili are present, as are OmpA-like proteins, which are associated with membrane stability and possible adhesion functions, and autotransporters (TAAs), which are involved in adhesion processes and possibly biofilm formation [88,89,90].

Biofilm formation in *P. salmonis* has been experimentally associated with the production of an extracellular polymeric substance (EPS) matrix of predominantly polysaccharide nature [48]. At the molecular level, genomic and functional analyses further support the existence of biosynthetic pathways for surface-associated polysaccharides, including lipopolysaccharide (LPS) and EPS, indicating that *P. salmonis* possesses the machinery required to produce and export these polymers. Importantly, EPS in Gram-negative bacteria can remain associated with the outer membrane as a capsule or be released into the extracellular environment, where it contributes directly to biofilm matrix formation [84]. This suggests that in *P. salmonis*, the cell envelope and its polysaccharide components are functionally linked to biofilm development, not only by providing structural elements at the cell surface but also by supplying extracellular polymers that mediate cell aggregation, surface attachment, and matrix stability.

The previous results show that ChS exhibits concentration-dependent antibiofilm activity. This effect is significant between 6 days and 8 days of exposure, but not in the early (4 days) exposure periods. This effect may be due to the interaction of ChS with the previously mentioned structures in the outer membrane of *P. salmonis*, which contribute to biofilm formation [48,84,85,86,87,90]. Therefore, this antibiofilm activity is likely associated with the results of this study regarding altered membrane permeability (Figure 5 and Figure 7) and structural modifications of the bacterial envelope (Figure 9). These changes may have contributed to the disorganization of EPS, weakening or modifying the biofilm matrix via ionic and non-ionic interactions with ChS.

Currently, no experimental evaluations have been reported on the effect of pure or modified chitosans on *P. salmonis* biofilm production. However, several important mechanisms of chitosan activity on bacterial biofilms have been distinguished in Gram-negative bacterial models (*E. coli* and *P. aeruginosa*), such as: Interference with bacterial communication strategies (quorum sensing), electrostatic attraction and degradation of the EPS matrix, advanced penetration facilitated by conjugation with specific ligands improving efficacy, even against mature biofilms, in addition to membrane permeabilization and direct antibacterial effects, according to the polycationic nature of chitosans [61,91,92,93,94].

Additionally, assays with chitosan modified with sulfur, but retaining its positive charge, demonstrated a dose-dependent reduction in biofilm formation in *P. aeruginosa,* ranging from 24.5% to 75% [57]. Other reports used chitosans modified with sulfur, which had a net negative charge, and describe the following: Biofilm inhibition with chitosan nanoparticles loaded with silver sulfadiazine in microbes isolated from wounds [95], significant antibiofilm activity with sulfonated low molecular weight chitosan against *E. coli* and *S. aureus* [96], inhibition of adhesion and biofilm formation with standardized sulfonated biomedical chitosan [97], as well as induction of bacterial death and consequent reduction in biofilm [98], both assays on *P. aeruginosa*.

Although these previous reports did not investigate *P. salmonis*, they indicate that sulfur modifications to ChC to change their net charge to negative can decrease or alter biofilm formation in bacteria. This supports the antibiofilm results of this study, where negatively charged chitosan significantly decreased biofilm formation in *P. salmonis* (Figure 10), ranging from 34.8% to 52%.

Regarding these results, it is worth mentioning that biofilm formation in this bacterium is a complex phenomenon and a virulence factor, and that most research has been conducted in vitro in *P. salmonis.* The form biofilms contribute to transmission and antimicrobial resistance through adaptive gene regulation, thereby ensuring their survival in the host [48,49,50,51].

Furthermore, environmental factors, such as NaCl concentration and iron availability, have been shown to significantly increase biofilm production across different strains of *P. salmonis* [99]. Consequently, strategies aimed at disrupting biofilm formation represent an important target for SRS control. The inhibitory effect observed for ChS at a MIC (1500 µg/mL) is consistent with previous reports describing the antimicrobial and antibiofilm properties of sulfur-modified chitosans. Interestingly, the lack of inhibition at 750 µg/mL of ChS (sub-MIC) after 4 days of exposure could reflect a phenomenon frequently observed with antimicrobial compounds, where sub-MIC levels can stimulate bacterial adhesion, biofilm formation (Figure 10), or stress responses, rather than inhibit them. In the case of *P. salmonis*, exposure to natural substances (phytogenic feed additives) at subinhibitory levels has been shown to modulate the expression of genes related to stress and biofilm formation, indicating that low concentrations of antimicrobials can activate adaptive responses in bacteria to increase biofilm formation [100].

This phenomenon has also been described for licensed antibiotics such as FF, where exposure to sub-MIC levels in *P. salmonis* influences biofilm formation and the expression of virulence genes, depending on the strain and surface material [10,51]. Other results demonstrated that sub-MIC dilutions of FF significantly modulated the expression of the efflux pump *acrAB* and two components of the system, *cpxAR* and *qseBC*, as well as florfenicol resistance genes (*tclor/tflor* and *t.flor*) in *P. salmonis* isolates embedded in analyzed biofilms [101]. Therefore, the increase in biofilm observed at 750 µg/mL in the present study probably reflects a stress-induced response that promotes bacterial adhesion or EPS production in the extracellular matrix. This background information underscores the importance of the proper management and responsible use of authorized antimicrobials and emerging antimicrobial alternatives.

The results obtained support the hypothesis that chemical modification of chitosan with sulfate groups generates a biomimetic HS receptor with greater affinity for the membranes of Gram-negative bacteria and increased capacity to destabilize the membrane of *P. salmonis* and reduce biofilm formation. Also, due to the lack of scientific background on the exposure of *P. salmonis* to ChS and other chitosans, the data obtained in this study represent the first results reported on the use of a biomimetic biopolymer of HS as an antibacterial alternative against this important pathogen of Chilean aquaculture.

Finally, due to the risk of bacterial resistance and the environmental impact associated with the frequent use of antimicrobials to treat and control SRS [4,8,11,101], in vivo studies and salmonid infection models are required to evaluate the therapeutic potential of these compounds. This includes their use in combination with approved antimicrobials (FF and OTC) for synergistic control, as well as their safety and biocompatibility assessment under aquaculture conditions. Overall, these findings improve our understanding of the structure-function relationships of modified chitosan derivatives, highlighting their potential role in developing antimicrobial alternatives.

## 4. Materials and Methods

### 4.1. Degree of Sulfation (DS) Estimation

The DS of ChS was estimated from elemental composition obtained by energy-dispersive X-ray spectroscopy (EDS). The calculation was based on the sulfur-to-nitrogen *(S/N*) ratio, assuming one amino group per glucosamine unit in the native chitosan structure, as previously reported [36], according to the following equation:

$$DS=\frac{\left(S/N\right)}{(S/N)+k}$$

where *S* and *N* represent the weight percentages of sulfur and nitrogen, respectively, obtained from the EDS analysis, and *k* is a proportionality constant derived from the molecular composition of the glucosamine monomer.

In addition, the DS was independently validated using a stoichiometric approach based on sulfur content, as previously reported [102,103]. This approach considers the molar mass of the chitosan repeating unit and the contribution of sulfate groups. Furthermore, the theoretical sulfur content (%S) was estimated from the DS values using mass-balance considerations for the glucosamine unit and sulfate substitution.

Elemental analysis using EDS revealed sulfur and nitrogen contents of 10.1 and 4.8 wt.-%, respectively, for the ChS sample. Based on these values, the DS was calculated using two independent approaches mentioned previously. In this context, the DS was determined to be 0.95 using the sulfur-to-nitrogen ratio method. Meanwhile, the molar ratio stoichiometric approach yielded a similar value of 0.92, confirming the consistency of the calculation. Theoretical sulfur content estimated from the calculated DS values ranged between 12.6% and 12.8%, which is slightly higher than the experimental value obtained by EDS (10.1%).

Elemental composition of the ChS was analyzed using energy-dispersive X-ray spectroscopy (EDS) coupled to the SEM system (Brand: JEOL, Model: JSM-IT300 Pleasanton, CA, USA). Spectra were obtained from multiple regions of each sample to determine the relative abundance of carbon, oxygen, nitrogen, and sulfur. The presence of sulfur confirmed the successful incorporation of sulfate groups into the chitosan structure. Because EDS provides semi-quantitative elemental measurements, the DS value obtained should be considered an approximate estimation of the substitution level.

### 4.2. Structural and Morphological Characterization of ChS

Fourier transform infrared spectroscopy (FTIR/ATR) of pulverized ChC and ChS samples was analyzed by using an Interspec 200-X instrument (Interspectrum OU, Toravere, Estonia). FTIR Spectra were recorded over a spectral range of 4000–400 cm^−1^ with a resolution of 4 cm^−1^. Characteristic absorption bands of ChC and the functionalized ChS were identified, including peaks at 1260 cm^−1^, corresponding to the asymmetric stretching of sulfate groups (S=O), and 800 cm^−1^, attributed to C–O–S vibrations. These signals confirmed the incorporation of sulfate groups into the chitosan backbone, mainly at the C2 and C6 positions of the glucosamine units.

### 4.3. Scanning Electron Microscopy (SEM) of Polymer

The polymer’s surface morphology was examined using scanning electron microscopy (SEM). Samples were mounted on aluminum stubs and coated with a thin gold–palladium layer using a sputter coater to improve conductivity. Images were acquired using a JEOL JSM-IT300 scanning electron microscope (CA, USA) operating at an accelerating voltage between 10 kV and 15 kV. Morphological features, including particle size, aggregation state, and surface roughness, were evaluated. The average particle size of the ChS ranged between 1 µm and 3 µm.

### 4.4. X-Ray Diffraction (XRD) of Polymer

X-ray diffraction (XRD) patterns of pulverized ChC and ChS samples were recorded using a D2 Phaser XRD system equipped with a Lynxeye detector (Bruker, Karlsruhe, Germany) and a Cu Kα radiation source (λ = 0.15406 nm) operating at 30 kV and 10 mA. A continuous scan was performed on an air-dried sample on a glass plate, with a step width of 0.02°, over 3–80° 2θ.

### 4.5. Biopolymer Preparation

ChS solutions were prepared at concentrations ranging from 31 µg/mL to 3000 µg/mL in sterile phosphate-buffered saline (PBS, pH 7.4). The ChS had a molecular weight of 70 kDa and a degree of deacetylation of >75% and a zeta potential (ζ) value of −22 mV [39]. The ChC used as a control had a molecular weight of 70 kDa and a degree of deacetylation of 75%, as determined by the manufacturer (ALDRICH^®^ San Diego, CA, USA), and was solubilized in a 1% acetic acid solution [35]. Sterile 10 mg/mL stock solutions of both biopolymers were prepared and filtered through 0.22 µm membranes (Startech^®^ London, ON, Canada). Given the importance of this variable in the antibacterial effect of these polymers, the pH of both the culture medium and the final solutions was measured.

### 4.6. Antibacterial Activity Assays

The antibacterial activity of ChS against *P. salmonis* was evaluated using the microdilution method described in CLSI M07 [104] (corresponding to the same methodology described in ISO 20776-1 [105]), with modifications adapted for slow-growing fish pathogens [1].

Bacterial Culture: The *P. salmonis* strain from genogroup LF-89 was donated by the Institute of Nutrition and Food Technology, University of Chile, Santiago, Chile. *P. salmonis* strain LF-89 was cultured in Austral-SRS medium prepared in the laboratory, as described by the authors and supplemented with 1% NaCl and 0.1% L-cysteine [106,107]. Cultures were incubated at 18 °C with gentle agitation (150 rpm) until reaching the logarithmic growth phase (OD630 ≈ 0.2). Bacterial suspensions were then adjusted to approximately (0.5 McFarland standard ~1.5 × 10^8^ CFU/mL) for all assays.

### 4.7. Minimum Inhibitory Concentration (MIC)

The MIC of ChS against *P. salmonis* was determined by broth microdilution. Serial two-fold dilutions (1:2) of ChS and commercial chitosan (ChC) were prepared. The concentration range tested in the assay was 46.9–6000 μg/mL.

Antimicrobial susceptibility assays were carried out in sterile 96-well microtiter plates. Each well contained: 155 μL of fresh Austral-SRS, 25 μL of the corresponding compound dilution, 20 μL of bacterial inoculum. The bacterial inoculum was adjusted to a 0.5 McFarland turbidity standard. The final volume in each well was 200 μL. The plates were incubated at 18 °C with constant agitation (150 rpm) for 144 h.

In addition to the evaluated polymers, reference antimicrobial agents commonly used for the treatment of piscirickettsiosis were included as comparative controls—specifically, oxytetracycline, florfenicol, and ChC were also tested under the same conditions to compare their antimicrobial activity with that of ChS. The following internal controls were included in each microplate: Growth control (culture medium + bacterial inoculum without antimicrobial compound), sterility control (culture medium without bacteria), and polymer blank (turbidity controls ChS and ChC without bacteria).

The MIC was initially determined by visual assessment of turbidity in wells and by an instrumental criterion, corrected optical density (OD). However, the final decision was based on microbiological evidence: volumes were re-inoculated from each evaluated well (according to CLSI recommendations when optical interferences are present) onto SRS medium (in duplicate). This method was adopted because previous observations showed a lack of viability upon re-inoculation in wells with corrected OD in the range of 0.05–0.10, indicating that a purely instrumental threshold (OD) did not accurately reflect actual bacterial viability. All experiments were performed in three independent biological replicates (n = 3).

### 4.8. Minimum Bactericidal Concentration (MBC)

To determine the minimum bactericidal concentration, aliquots from wells showing no visible growth were reseeded in Austral-SRS medium and incubated for 14 days at 18 °C with constant agitation (150 rpm), to confirm the absence of bacterial growth during the incubation period (14 days), as suggested by previous reports [1] on *P. salmonis* due to its slow in vitro growth kinetics. The MBC was defined as the lowest concentration at which no bacterial colonies (CFU) were observed.

### 4.9. Biofilm Formation Inhibition Assay

Biofilm formation by *P. salmonis* exposed to ChS was evaluated using the protocol described by Santibañez et al. [99], with some modifications. The bacteria were cultured in sterile, 96-well, flat-bottom polystyrene microplates (JET BIOFIL, Guangzhou, China). Austral-SRS medium was used, prepared in the same manner as for MIC determination in this study. All wells had a final volume of 200 µL, prepared as follows: each control inoculum well was first filled with 180 µL of sterile Austral-SRS medium. Meanwhile, the Ch-S treatment wells were filled with 180 µL of a solution containing two Ch-S concentrations: the MIC (1500 µg/mL) and a sub-MIC (750 µg/mL), using the same sterile Austral-SRS medium as the solvent.

Similarly, the control antibiotic (oxytetracycline) was added to the corresponding wells in a solution volume of 180 µL, at a concentration of 5 µg/mL (slightly higher than the MIC of the bacterial strain (3.9 µg/mL) to provide positive antibiofilm control. Once all the microplates contained the volumes described above, the volume was brought up to 200 µL with 20 µL of bacterial inoculum (0.5 McFarland standard ~1.5 × 10^8^ CFU/mL) per well.

In addition, sterile Austral-SRS broth (200 µL) and ChS solution (200 µL) without inoculum at the two experimental concentrations (750 and 1500 µg/mL) were used as negative controls and to correct the OD_blanks measurements due to the increased background signal caused by the nonspecific interaction of the CV with polymeric matrices [108,109] like ChS. The microplates were capped, and the edges were covered with permeable parafilm. They were incubated at 18 °C without shaking for up to 8 days.

Biofilm formation was quantified at 4, 6, and 8 days of culture by crystal violet (CV) staining, following a standardized protocol [99,110] with modifications. The volume of each well was discarded, and the plates were washed with 1× PBS by gentle pipetting. The plates were then allowed to dry at 37 °C for 30 min. Two hundred microliters of CV 0.1% were added to each well, and after 15 min of incubation, the CV solution was discarded. The wells were washed with sterile distilled water by slow, repeated pipetting to remove excess CV staining.

Next, they were dried at room temperature in an inverted position for 30 min. Then, the CV was solubilized by adding 200 µL of 96% ethanol for 10 min, and the absorbance was measured at 595 nm using a microplate reader (BIO-RAD model 3550, Hercules, CA, USA). Biofilm production was calculated according to the method of Stepanović et al. [110], using a proposed OD cutoff value (DOc). DOc is defined as three standard deviations (SD) above the mean OD of the negative control (sterile medium): DOc = DOn + 3 × SD. DOn is the mean OD of the negative control, and SD is its standard deviation. Therefore, the OD value of an analyzed strain will be expressed as the mean OD of the strain or condition, minus the DOc value. The DOc value will be calculated separately for each microtiter plate. A negative value will be represented as zero (0), while any positive value will indicate biofilm production. The percentage of biofilm inhibition was calculated using the equation:

$$\mathrm{Biofilm}\;\mathrm{inhibition}\;(\%)=\left(1-\frac{A_{\mathrm{treated}}}{A_{\mathrm{control}}}\right)\times 100$$

All experiments were performed in three independent biological replicates.

### 4.10. Bacterial Viability and Membrane Permeability by Fluorescence Microscopy (LIVE/DEAD Imaging)

Membrane integrity of *P. salmonis* cells exposed to ChS was evaluated using the LIVE/DEAD BacLight bacterial viability kit (Thermo Fisher Scientific, Waltham, MA, USA). Bacterial suspensions (0.5 McFarland standard ~1.5 × 10^8^ CFU/mL) were exposed to ChS at its MIC (1500 µg/mL) and the same culture without polymers was used as a negative control, and ethanol at 50% concentration was used with the culture as a positive control for rapid membrane permeabilization, as it is known to disrupt bacterial membrane structure, increase membrane fluidity, and induce leakage of intracellular contents, leading to loss of membrane integrity and uptake of membrane-impermeable dyes such as propidium iodide [111] and incubated at 18 °C with agitation (150 rpm). Samples were stained with SYTO9 (6 µM) and PI (30 µM) according to the manufacturer’s instructions and previous reports [90]. Samples were taken for vital staining at 10 min, 30 min, 1 h, 5 h, 24 h, 48 h, 72 h, 96 h, and 120 h of experimental exposure to the polymers and controls. Following experimental exposure to the biopolymers, the samples analyzed using the fluorescence ratio assay were subsequently cultured in Austral-SRS medium to evaluate bacterial recovery after 120 h of incubation with the treatments.

Fluorescence images were obtained using a Zeiss LSM 800 confocal laser scanning microscope (Oberkochen, Germany) with excitation wavelengths of 488 nm (SYTO9) and 561 nm (PI). Image analysis was performed using ImageJ software (Version 5.4) to quantify fluorescence intensity. Green fluorescence corresponded to cells with intact membranes, while red fluorescence indicated compromised membranes. Changes in bacterial cell membrane permeability were expressed as the Green/Red fluorescence ratio, calculated from the average of three experimental replicates.

Images correspond to independent fields obtained from separate samples at each time point. Post-acquisition image processing and merging were performed using Fiji (ImageJ distribution, Fiji Is Just). Individual green and red channel images were imported into the software and combined using the Merge Channels function to generate composite (overlay) images. Merged images (Green/Red overlays) were used to enable accurate visualization of membrane integrity and signal co-localization within the same field. Representative images were selected from replicate experiments to illustrate consistent patterns observed across independent experiments.

### 4.11. Scanning Electron Microscopy (SEM) of Bacteria

Morphological analysis was performed using a JEOL JSM-IT300LV microscope (Pleasanton, CA, USA) located at the Faculty of Dentistry of the University of Chile. To examine changes in bacterial morphology by SEM, samples were collected from *P. salmonis* cultures exposed to ChS and ChC, and from a control culture without polymer exposure. Before evaluation, the samples were lyophilized and metallized with gold, as previously described [112].

### 4.12. Statistical Analysis

All experiments were performed in triplicate. Results are expressed as mean ± standard deviation (SD), and, for corrected OD in biofilm measurement tests, the standard error of the mean was used. Statistical significance between treatments was determined using one-way analysis of variance (ANOVA) followed by Tukey’s post hoc test using GraphPad Prism version 9.0. Differences were considered statistically significant when *p* < 0.05.

## 5. Conclusions

Sulfated chitosan (ChS) exhibited significant antibacterial activity against *Piscirickettsia salmonis*, representing the first evidence of its effect on bacterial membrane permeability as a heparan sulfate-mimetic polymer. SEM-EDS confirmed a high degree of sulfation (0.92–0.95), while XRD analysis demonstrated a reduction in crystallinity, indicating a more amorphous structure with enhanced flexibility and accessibility of functional groups.

These structural changes, together with increased charge density, likely explain the stronger interaction of ChS with bacterial membranes and its ability to induce progressive permeability disruption, as confirmed by fluorescence assays and microscopy. SEM analysis further revealed severe morphological damage and aggregation, while biofilm formation was significantly reduced (*p* < 0.05) at prolonged exposure times.

Overall, the combined effects of reduced crystallinity, increased charge density, and biomimetic properties support the role of ChS as an effective antimicrobial and antibiofilm agent. Its biodegradable and biocompatible nature highlights its potential as a sustainable alternative to reduce antibiotic use in aquaculture within a One Health framework.

## Figures and Tables

**Table 1:** Relative potency compared to the MIC of ChS, vs. ChC, OTA, and FF against *P. salmonis* strains LF-89.

Treatment	MIC (µg/mL)	MBC (µg/mL)	MBC (µg/mL)
ChS	1500	≥1500	Relative potency compared to the MIC of ChS
ChC	3000	≥3000	2 times less potent
OTA	3.9	≥3.9	~385 times more potent
FF	4	≥4	~375 times more potent

MBC: minimum bactericidal concentration; MIC: minimum inhibitory concentration.

## Data Availability

The data presented in this study are available on request from the corresponding author.
